# Multi-foci laser microfabrication of 3D polymeric scaffolds for stem cell expansion in regenerative medicine

**DOI:** 10.1038/s41598-019-48080-w

**Published:** 2019-08-13

**Authors:** Tommaso Zandrini, Oumin Shan, Valentina Parodi, Giulio Cerullo, Manuela T. Raimondi, Roberto Osellame

**Affiliations:** 1 Politecnico di Milano, Department of Chemistry, Materials, and Chemical Engineering “Giulio Natta”, Milano, 20133 Italy; 2 National Research Council, Institute for Photonics and Nanotechnologies, Milano, 20133 Italy; 3 Politecnico di Milano, Department of Physics, Milano, 20133 Italy

**Keywords:** Regenerative medicine, Laser material processing

## Abstract

High quality large scale fabrication of cellular scaffolds, with three-dimensional resolution comparable to cell size, is an important task to enable regenerative medicine applications with stem cells. We are using two-photon polymerization to produce our stem cell culture substrate called Nichoid, which we already demonstrated capable of stimulating cell proliferation while maintaining their stemness, without the need of dangerous additives. Parallelization of this technique can be achieved with the use of a spatial light modulator: here we show the results obtained combining this device with fast linear stages to produce Nichoid-covered substrates by two-photon polymerization. The well-polymerized structures confirm that this approach is particularly convenient for porous structures, and allows a significant time saving by a factor of almost five, with minor design adjustments. A Live & Dead assay was performed on mesenchymal stem cells cultured into the Nichoid microstructures in order to verify that no difference in cell viability is present, compared to microstructures fabricated by a single focus. This parallel setup opens the possibility to obtain a much larger number of microstructured substrates, that are essential to test new stem cell-based therapies. This approach can be also used for the fast fabrication of other kinds of cell culture devices.

## Introduction

Polymeric scaffolds are an enabling tool for cell culture, since they create a three-dimensional (3D) environment that can influence cell growth and proliferation. In particular, stem cell culture on scaffolds is of great interest, as it offers the possibility to control, stimulate or prevent the differentiation of such cells towards certain types, depending on mechanical and chemical stimuli^1–7^. Feeder layers and other biological compounds, such as decellularized matrices^8^, foetal bovine serum, Leukemia inhibitory factor, or Matrigel, are usually employed in stem cell expansion *in vitro* systems^9^, due to their simplicity and effectiveness in maintaining pluripotency; however, concerns have been raised regarding their xenogeneic (i.e. derived from another species) nature^10^. Alternative xeno-free products (not derived from other species) are beginning to appear on the market^10,11^, but they are still considered unsafe for diagnostic and therapeutic use by the producers. Moreover, the large number of available methods, substrates, and chemical and biological additives for stem cell expansion makes a rigorous quality control under good manufacturing practices (GMP) conditions in cell factory facilities very challenging^9,12^. This is crucial to ensure reproducibility of results and safety of procedures, to upgrade the process from laboratory results to clinical studies and therapeutic applications on human patients. To avoid any kind of influence from external compounds that can prevent clinical applications, we developed a 3D polymeric biocompatible scaffold, that has been shown capable of maintaining the function of both pluripotent and multipotent stem cells with only mechanical stimuli^13^. This microstructured substrate, called “Nichoid” is fabricated through the direct laser writing technique known as two-photon polymerization (2PP)^14^. The main limitation of our approach is the serial nature of 2PP, which hampers the production of a large number of samples with high control on their geometry and in a short time. This large number of samples is essential for the expansion of the great quantity of cells needed in biological research projects, where tens of substrates are needed to have sufficient statistics and controls, and in clinical applications^15^. In order to increase the throughput of 2PP, many research groups worked on parallelization techniques to multiply the number of simultaneous features fabricated on the same sample. The first works on this topic made use of beamsplitters or microlens arrays to increase the number of polymerizing spots focused inside the photoresist^16,17^. Although this method is very straightforward, it lacks the capability of quickly reconfiguring the spots pattern. More recent works introduced a spatial light modulator (SLM) into the laser fabrication setup^18–22^. SLMs are liquid crystal diffractive devices in which the pixel matrix can impose a custom phase to the impinging beam, resulting in a precise intensity pattern in the far field, i.e. in the focus of the fabrication objective. The easy reconfigurability of these devices allows for on-the-fly change of the foci pattern during the fabrication process with a frame rate typical of computer screens.

Kelemen *et al*. and Takahashi *et al*. reported fabrication velocities between 25 and 100 µm/s, using piezoelectric translators^18,19^. The groups of Gittard *et al*. and Yang *et al*. instead, took advantage of the very high translation speed allowed by galvanometric scanners^20,21^. The first group reported a fabrication speed of 20 mm/s with single focus, and 2.5 mm/s with the multi-foci configuration; notwithstanding the lower speed, the use of multiple foci allowed a time saving factor of 2.5. The second group claims a time saving factor of 3.3 with the SLM with respect to the single-focus setup.

Galvanometric scanners are the fastest option currently available on the market for beam translation, but they suffer from a few limitations: first, the movement of the laser focus is limited to the plane perpendicular to the laser beam, so that the vertical translation is always performed through a linear translator at a lower speed. Second, the accessible area is limited to the field of view of the used objective, which is typically between 100 and 300 µm for high magnification objectives, decreasing when magnification and numerical aperture increase. This means that a scanner can only trace lines on a single plane, and with a limited length, so that it is very convenient for bulky structures, where a plane by plane irradiation approach is the most suitable one, and the stitching of various parts of a larger structure is possible.

Other groups tried to increase the throughput and therefore the size of the fabricated structures by regulating the voxel size, for example by controlling the focal spot size through either spatiotemporal focusing^23^, or the use of low NA objectives with adequate aberration compensation^24,25^, or alternating different NA objectives depending on the required resolution^26^. Voxel size regulation is again very effective for large structures with bulky regions, but less convenient when a small voxel size is required throughout the whole fabricated structure.

A different approach consists in avoiding both sample translation and beam scanning, by using only the SLM capability to control the light intensity profile after the objective lens. Multiple spots can be moved in a synchronized fashion to irradiate, plane by plane, three-dimensional structures. The calculation of the holograms can be performed on the fly^27^, or in advance^28^. This approach avoids any inertia issue due to the translation mechanisms, but is limited to the objective’s field of view, and suffers from limited scanning velocity, due to the limited refresh rate of SLMs. A similar technique has been recently demonstrated using digital micromirrors instead of SLMs, with a much higher refresh rate^29^.

Multiple spots are not the only solution for parallel scanning: a complex image can be projected at once, provided that the impinging laser power is sufficient to achieve polymerization^30^. In their work, Zhang and coworkers employed a more efficient hologram generation algorithm, rather than the most widespread Gerchberg-Saxton. This technique greatly accelerates the patterning process for two-dimensional textures but is reported to be less suitable for three-dimensional fabrications, since it guarantees a better control on the focal plane, but a poor one outside of it.

For very porous structures, with a large number of vertical lines and long and thin horizontal lines, such as our Nichoid scaffold, galvanometric scanners are not convenient compared to fast linear stages: they would have to scan each vertical line plane by plane instead of performing a single vertical movement, and, in case of stitching, long lines should be connected with extreme precision to avoid breaking. We already published our results on the up-scaling of Nichoid scaffold fabrication over a large area with linear translators^31^, and recently another work on the same topic with notable results came out, confirming the effectiveness of this approach for this kind of structures^32^.

To combine the advantages of the various approaches, we decided to upgrade our linear stages fabrication system with a spatial light modulator, in order to increase the fabrication speed. We chose to focus on the Nichoid not only because it is a complex 3D structure archetypal of a whole class of cell scaffolds, but also because this specific structure is very relevant in stem cells culture, as we have demonstrated^13,33^ and are further investigating. The aim of this work is to establish the benefits of this approach, compare the quality of the structures polymerized with multiple foci with the single focus ones, and verify that cells behaviour in the new structures is not affected by the polymerization technique upgrade, to confirm that they can be used for further biological studies.

## Results

### Multi-foci fabrication results

**Figure 1:**
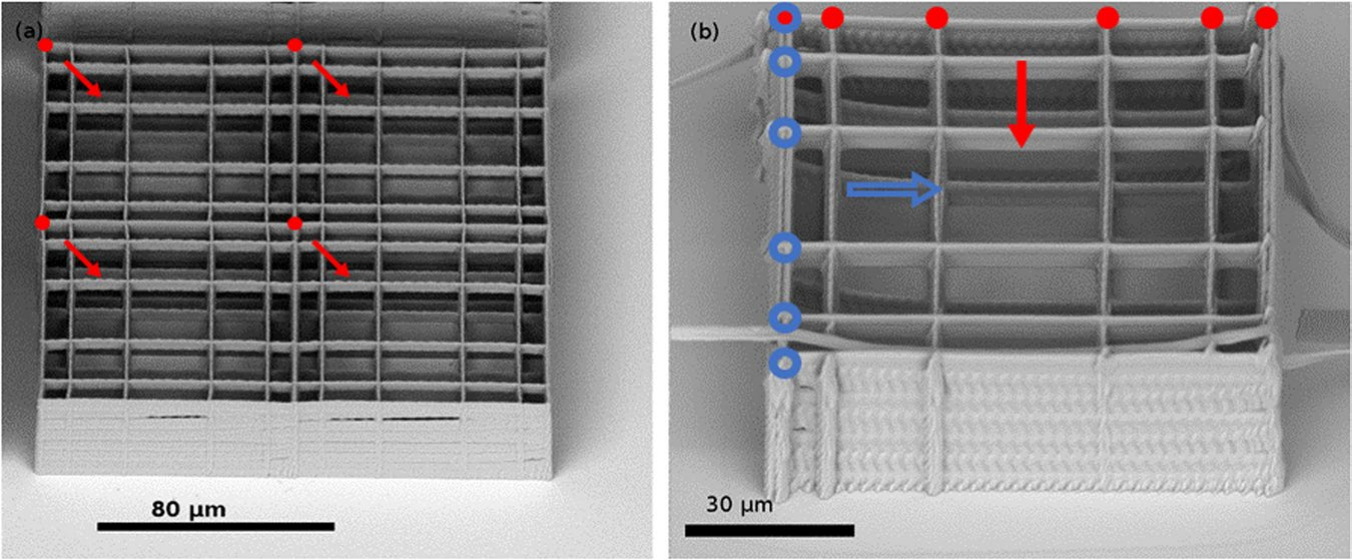
Nichoids realized with multiple foci. (**a**) 4 Nichoids fabricated simultaneously, with 4 foci. Each red dot represents one of the foci used to build the whole single Nichoid. The arrows indicate the scaffold fabricated by each dot. (**b**) One Nichoid produced with 6 and 2 foci, oriented along two perpendicular directions. Red dots and blue dots represent the two 6-foci patterns used to scan the grids along the two perpendicular directions. One of the two patterns has also been used to fabricate the columns, six at a time. 2 foci patterns are constituted by the two external dots of the 6 foci ones and were used for the side walls. Here the arrows indicate the scanning direction of each pattern. Fabrication parameters are reported in Table 1.

We could obtain well-polymerized single Nichoids both with 4 static foci, and with dynamically changed 6 and 2 foci masks, as reported in Fig. 1. The 4 Nichoids took 17 s to be fabricated, which means that we can consider an effective fabrication time per Nichoid of 4.25 s. The 6 foci Nichoid was irradiated in 6 s. Complete fabrication data are shown in Table 1.

**Table 1:** Scaffold fabrication parameters with the same laser source.

Parameters	1-focus Nichoid	4-foci Nichoid (Figure 1a)	6-foci Nichoid (Figure 1b)	1-focus Nichoid blocks (Figure 2a)	6-foci Nichoid blocks (Figure 2b)
Number of Nichoids	1	4	1	5450	5450
Single Nichoid fabrication time	17 s	4.25 s	6 s	7 s	1.5 s
Total fabrication time	17 s	17 s	6 s	12 h	2.5 h
Average total laser power	21 mW	107 mW	260 mW	21 mW	260 mW
Peak intensity on sample per focus	7.2 TW/cm^2^	9.2 TW/cm^2^	15 TW/cm^2^	7.2 TW/cm^2^	15 TW/cm^2^
Energy dose per unit area	2.1 MJ/cm^2^	2.7 MJ/cm^2^	4.5 MJ/cm^2^	2.1 MJ/cm^2^	4.5 MJ/cm^2^
Translation speed	3 mm/s				

The 1-focus data are compared to the ones obtained with the 4 and 6 foci configuration performed with the use of the SLM, both for single Nichoids and for substrates covered by Nichoid blocks. Laser power has been measured before the focusing objective. Peak intensity on sample has been calculated for each focus with the parameters reported in the Materials and Methods section according to the formula provided in ref.^37^. The total fabrication times for large samples measured are slightly higher than the ones that can be calculated from the table, because they include the travel time from one block to the following one.

**Figure 2:**
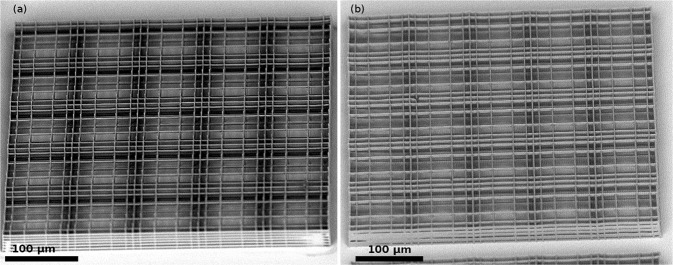
5 × 5 Nichoids block, realized with multiple laser foci. (**a**) 5 × 5 Nichoids block, polymerized with a single laser focus. (**b**) 5 × 5 Nichoids block, polymerized with 6 foci for the vertical columns and grid lines, and with 2 foci for the walls separating each Nichoid. Fabrication parameters are reported in Table 1.

We also realized a complete Nichoid block, constituted by 5 × 5 Nichoids, with the 6-foci configuration, in 38 s. We managed to cover a 4 mm radius area on a 12 mm diameter round glass coverslip with the Nichoid microstructures, organized in blocks. Thanks to the long travel linear stages, it was possible to use the same foci configuration to polymerize a set of lines of the whole Nichoid block in a single scan, thus saving time with respect to the single Nichoid fabrication, since we avoid many acceleration/deceleration steps and mask changes. With this technique, combined to a laser scanning path designed to reduce the mask changes and minimize out of plane movements, we could reduce the fabrication time to 1.5 s per Nichoid, as it appears from the data in Table 1. The only issue appearing from a scanning electron microscope (SEM) inspection of the structure, shown in Fig. 2, is the thickness of the lines polymerized with multiple foci, which is larger than that of the single focus ones. The reason is that the laser beam profile generated by the SLM at the objective aperture to create a multifoci pattern is not exploiting the full numerical aperture of the objective (see Materials and Methods), as a properly expanded Gaussian beam usually does, and therefore the resolution of the writing process is reduced, mainly in the depth where the confocal parameter is more sensitive to the numerical aperture. Moreover, aberrations can be induced by the imperfect flatness of the SLM screen and by the numerically generated hologram on a finite number of pixels. These technical limitations could be overcome by an SLM with a larger number of pixels and withstanding higher power, thus allowing to overfill the objective and still have enough power for multi-foci fabrication.

**Figure 3:**
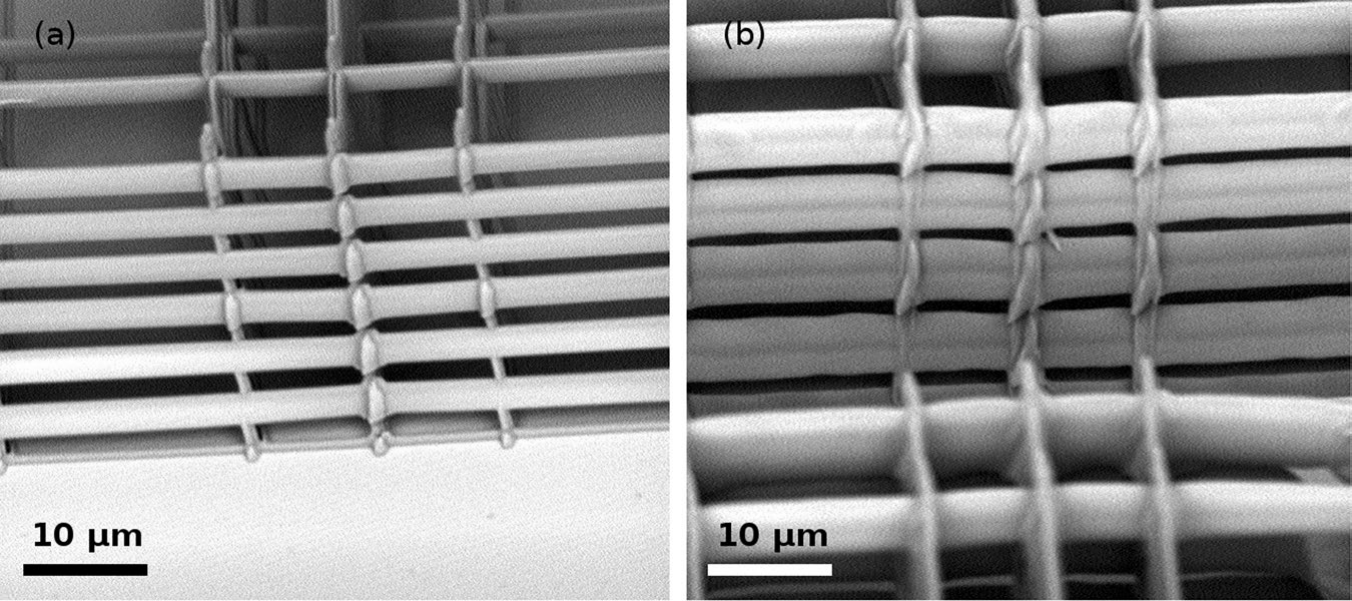
Lines from Nichoids block polymerized with 1 single focus (**a**) and multiple foci (**b**). Single focus lines are 3.0 ± 0.2 µm thick and well separated in the walls, while multiple foci lines are 6.9 ± 0.7 µm and leave smaller apertures. Fabrication parameters are reported in Table 1.

We observed that the apertures in the side walls of the Nichoids, useful to let nutrients diffuse into the scaffolds once they are fully colonized by cells, were heavily reduced in the scaffolds fabricated by SLM. To restore the same aperture width that was achieved with single-focus writing, we thus decreased the number of lines in the side walls, passing from 5 to 7. To quantify the thickness difference between the two polymerization techniques, we measured the thickness of almost one hundred lines belonging to different Nichoid blocks fabricated with multiple foci, and the average thickness resulted 6.9 ± 0.7 µm, while the average thickness for single-focus lines was 3.0 ± 0.2 µm. The difference between the lines in the two cases is visible in Fig. 3.

If in the single focus design the wall lines were distributed every 5 µm, always leaving clear spaces between them, in the multiple foci case their number has been reduced and the lines have been placed at a 7.5-µm distance one from the other, producing again an aperture between them.

### Autofluorescence measurements

The intrinsic fluorescence emission of the engineered scaffold was measured in the three main emission channels used in fluorescence microscopy: blue, green and red.

By summing the mean autofluorescence intensities of the grids in each stack, we observed that the Nichoid autofluorescence is present in every channel with different intensities. Calculating the ratio between the 1-focus and 6-foci scaffold autofluorescence, it resulted that the 1-foci scaffolds gave 74% of the intensity of the 6-foci ones in the blue channel, and 76% in the green one. For both Nichoids, the red channel did not show a signal above the noise.

### Biocompatibility test

**Figure 4:**
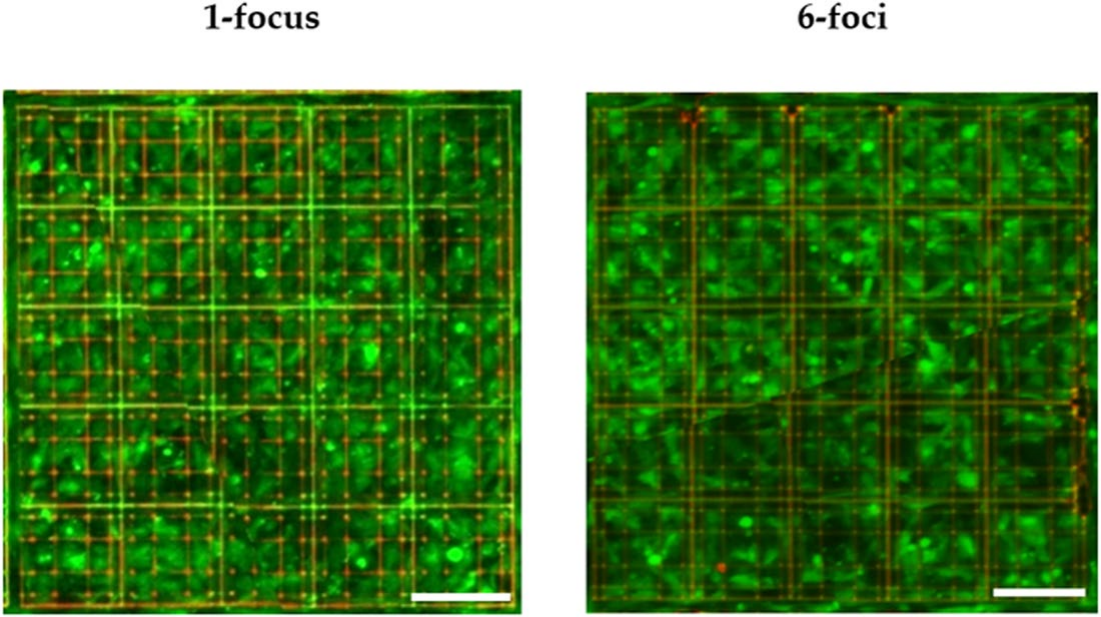
Z-stack projections of confocal images acquired in a Live & Dead assay on MSCs 72 h after seeding. On the left MSCs seeded in scaffolds fabricated through a single focus laser system, while, on the right, MSCs cultured in Nichoid polymerized through the 6-foci laser system. Green cells are alive while dead cells appear red; in evidence the auto-fluorescence of the scaffold in the red channel. The scale bar is 90 μm.

Our purpose was to validate the efficacy on mesenchymal stem cells viability of the Nichoid culture system fabricated with the SLM- equipped laser setup, as compared to the Nichoids fabricated with standard single-focus setup. This test was performed in order to exclude that small geometric differences or a different degree of crosslinking, that can be induced by a change in irradiation conditions^34^, could affect cell behaviour. To investigate cell response to Nichoids, we performed a Live & Dead assay on MSCs. Firstly, we evaluated cell viability on Nichoids produced by a single focus as a control experiment, then we repeated the procedure for a sample fabricated by the multi-foci SLM set-up. As clearly visible in Fig. 4, the Z-stack projection of the merged images in the three days of observation showed, for both cases, a high intensity green (Live) signal compared to the red (Dead) one, meaning that MSCs cultured in Nichoid 3D scaffolds fabricated by the multi-foci approach were for the majority alive.

**Figure 5:**
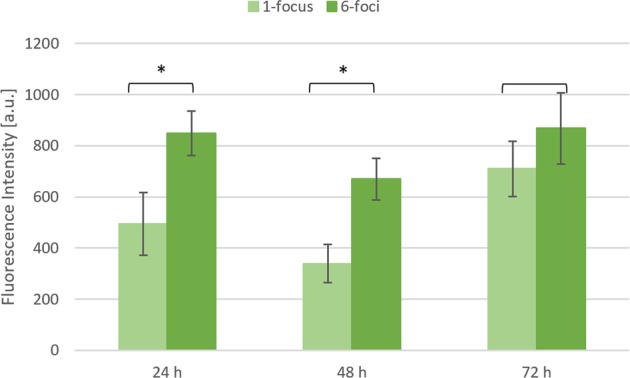
Viability of mesenchymal stem cells in culture in Nichoid substrates fabricated through two different setups during three days of observation (1-focus vs 6-foci). Data are given as mean ± standard deviation of twenty-five independent measurements with *p < 0.05 from pair-wise comparison of sample means.

The average fluorescence intensity values for each experimental image, obtained after background noise removal and single channels analysis, are represented in Fig. 5. Viability follows the same trend in the two populations, decreasing at 48 hours and increasing at 72 h. Moreover, cells seeded in Nichoids made with the use of the SLM show 20% more green fluorescence intensity emission with respect to the ones in culture in single focus-fabricated Nichoids. We performed the Student T-test to verify that the p-value was less than 0.05 among the two populations at each time point. The test revealed that the two populations were significantly different for the first 48 hours (p-values < 10^−8^), while they became statistically indistinguishable 72 hours after seeding (p-values > 0.2).

Furthermore, we measured a very low signal from the red channel, meaning that above the auto-fluorescence signal coming from the scaffold itself, few dead cells were present, therefore neither the single-focus Nichoid nor the 6-foci one induced cell death at any time point. MSCs grew properly, showing an increasing trend of green fluorescence intensity in time that can be related to stem cell viability and numerosity. Furthermore, a reduction of the green signal gap between the 1-focus and 6-foci culture systems was measured over time, passing from 42% at 24 hours to 19% at 72 hours.

## Discussion

The production of a large number of scaffold samples with high reproducibility is a key asset for their use in a high-quality research process under GMP. Self-assembly fabrications techniques are widely used for the low-cost and fast production of cell culture scaffolds, but, as we already discussed in previous works^13,31^, they lack the ability to finely control the geometrical features of the scaffold at the cell scale. The standard industrial technique of UV photolithography is unable to produce complex 3D structures in a single passage, while replica moulding has very limited capabilities of reproducing structures with voids and passing holes, even though some attempts have been made^35^.

The combination of galvanometric scanners with SLMs is not suitable for large and very porous structures such as the Nichoid, because the layer-by-layer approach is very time-consuming for its geometry. Foci translation through SLM suffers from the same defect, and it is unable to reach high velocities, while whole image projection gives poor control over the third dimension and is again a layer-by-layer approach.

With the fabrication technique illustrated in this paper we showed how we could speed up the polymerization of our Nichoid-covered substrate. Producing a single Nichoid with a static 4-foci approach turned out to be more time-saving than producing the same Nichoid with 6 and 2-foci patterns, mainly because the time required to switch between the different masks, longer than 0.5 s, was too relevant, compared to the laser scanning time. As it can be read in Table 1, producing the whole Nichoid block with single focus is already faster than irradiating Nichoids one at a time with the same setup, and only a few seconds slower than the multi-foci single-Nichoid approaches. By parallelizing Nichoid blocks irradiation with 6 and 2-foci patterns, we could further reduce this time by a factor of almost 5 compared to single focus, being almost 3 times faster than with the 4-foci static configuration.

Thicker lines resulted from SEM inspection, as visible in Fig. 3, due to the different optical quality of the multiple foci: the best focus quality obtainable from a high magnification objective, is achieved with a uniform illumination of its aperture. A less tight focusing of the spots generated by the first diffraction order of the SLM, which is the one exploited for fabrications, increases the power per focus needed to trigger good quality 2PP at the translation velocity of 3 mm/s. From Table 1 it is possible to see that the power needed for the six foci is 260 mW, which is more than 40 mW per focus, twice the power required by the single focus, 21 mW. As it is known from the literature, an increase in the power used for 2PP corresponds to a great elongation of the polymerized voxel along the laser beam propagation direction, resulting in thicker lines.

The structure stability was not affected by the different resolution, and a slight design change allowed to maintain the apertures on the side and internal walls, to help the diffusion of cell nutrients. The number of foci employed to fabricate the Nichoid was chosen taking into account the objective field of view, the symmetry of the structure, and the damage threshold of the SLM. A higher number of foci, at least double for symmetry reasons, would have been possible, but it would have required more than 1 W impinging on the SLM. At this power, during our preliminary studies, we observed a significant drift of the diffracted power over time, which means, according to the producers, that a prolonged exposure of the liquid crystal screen could lead to thermal damage. Other works reported use of an SLM for fabrications with a high foci number, but in all cases they had to decrease the laser scanning velocity, reducing considerably the time gain introduced by the multiplication of the focal spots, particularly for our kind of structure^20,21^.

The autofluorescence studies showed the direct link between the intrinsic fluorescence of the Nichoid and the amount of polymerized material. We obtained that the sum of the autofluorescence intensities of the 6-foci Nichoids was higher both in the blue and in the green channel than the one measured for the single focus. This was expected, since the multiple foci fabrication involved a larger volume of polymerized resist because of the larger voxel, compared to the standard single beam technique, as discussed earlier.

The Live & Dead assay showed that the change of fabrication approach did not hinder at all the capability of Nichoids to promote cell adhesion and proliferation. We can conclude that the changes in voxel size and crosslinking degree did not exert any harmful effect on cells, so our structures can be safely employed for cell culture experiments. By monitoring cell growth for three days with a non-destructive fluorescence approach we measured an increasing viability signal in time, meaning that cells proliferated efficiently. This signal in the sample produced with the SLM system was higher than the one obtained from the control scaffold produced with the single focus laser system, even though this difference became statistically insignificant on the third day of observation. We assumed that thicker lines could have helped cell adhesion during the first day after seeding, because cells would find a larger surface on which they could form focal adhesions, mediated by the proteins found in the culture medium. On the third day the cells reached confluence and were properly adhering to the scaffold’s surface, therefore the difference between the two populations became negligible.

In conclusion, we showed that the introduction of a spatial light modulator in a fast-linear stages two-photon polymerization setup is a viable and efficient strategy to produce a microstructured and highly porous cell culture substrate over a large area, with no observed drawbacks during cell culture. We have specifically discussed the use of SLM for the fabrication of the Nichoid because it is an excellent example of a highly porous, regular and extended structure, features that are typical for cell scaffold applications. Porous structures have been seldom discussed in terms of fabrication speed improvement, as usually very dense structures are considered. This study will open interesting possibilities in the field of regenerative medicine, where a large number of cells is needed to obtain a therapeutic effect, especially for the test of new therapies based on stem cell products. It is our intention to proceed in this direction, with more detailed biological experiments on the cells produced in our structures and on their capabilities. This method is also valid and can be applied to fabricate any kind of porous scaffold for cell culture, with geometries different from the Nichoid, or used for different cellular lines, or to induce a differentiation rather than preventing it, or even to realize implantable devices for *in vivo* applications, in a reduced fabrication time compatible with a technology transfer of the scaffold towards industrial production.

## Materials and Methods

The polymeric material used for our 2PP fabrications is SZ2080, a photoresist specifically designed for 2PP^36^. A commercial photoinitiator, Irgacure-369 (2-Benzyl-2-dimethylamino-1-(4-morpholinophenyl)-butanone-1), was mixed with the photoresist to increase the two-photon absorption cross-section and obtain a more efficient fabrication process. This material has been already used in previous works, where it has shown no cytotoxicity and a low autofluorescence, which are ideal conditions for cell culture and fluorescence microscope observations^33^.

**Figure 6:**
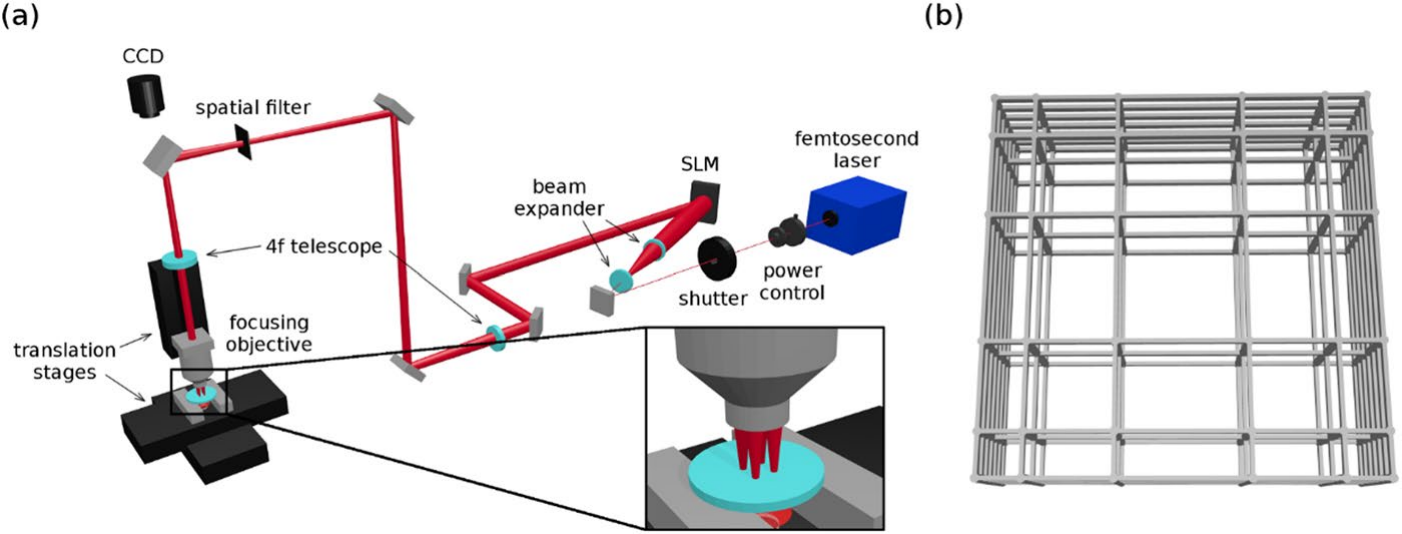
(**a**) Multi-foci laser polymerization setup. It is composed by the femtosecond laser source, a power control stage, a shutter unit and a microscope objective, whose focus can be moved in three dimensions with respect to the sample by fast linear translation stages. To insert the SLM into the setup, a beam expander and a 4f telescope have been added. A depiction of multi-foci illumination after the magnification objective is shown in the inset. (**b**) CAD drawing of a Nichoid. The pores have variable size, ranging from 10 to 30 µm side, and the grids are vertically spaced by 15 µm.

The laser polymerization setup (Fig. 6a) is equipped with fast computer-controlled linear translators with several cm of travel range (ANT-130, Aerotech), a manual power control stage consisting of a rotating half-waveplate and a polarizer, a computer controlled mechanical shutter to completely block the beam (LS6, Uniblitz), and a high magnification oil-immersion microscope objective (100x, 1.4 numerical aperture, 37% transmission at 1042 nm) to focus the laser beam inside the sample. The laser source employed for the fabrications presented in this work is a commercial femtosecond laser, with maximum power up to 8 W, pulses shorter than 400 fs, wavelength of 1042 nm, and 1 MHz repetition rate (femtoRegen, HighQ laser). The laser M^2^ factor is equal to 1.2, and the spot radius after the microscope objective is calculated to be 450 nm.

The spatial light modulator (SLM) that we inserted into the fabrication setup is a liquid crystal on silicon (LCOS) reflective SLM that performs phase only modulation (PLUTO NIR-049, Holoeye). To add it to the setup, we had to place also a beam expander, to illuminate the full screen with the laser beam, and a 4-f telescope between the SLM and the focusing objective. This was needed to make the beam size match the objective aperture, and to be able to perform spatial filtering of the zeroth diffraction order of the SLM, which is more powerful than the other desired foci and would hamper the fabrication. The spatial filter consisted in a plate with two perpendicular slits, to allow only the passage of the first order of diffraction from the SLM. We observed no polymerization caused by any ghost image. Phase masks for the multi-foci pattern generation have been calculated through the Holoeye software provided with the SLM, which uses the iterative Gerchberg-Saxton algorithm. The diffraction efficiency of the generated mask depends on the initial guess. In our case, we generated masks with a set of random guesses, and chose the most efficient one, after testing them in the optical setup. At this point, no correction of the wavefront has been implemented. We note that, due to limitation of the power that can be directed on the SLM screen without inducing drifts in the phase map (about 0.75 W/cm^2^, without any additional cooling system), we designed the 4-f telescope in order to have the rectangular image of the SLM screen inscribed inside the circular aperture of the objective. In this way we are able to use the whole laser power coming from the SLM, which is required for the fabrication with 6 foci, albeit slightly underfilling the objective aperture and thus not fully exploiting its numerical aperture.

In order to recreate irradiation conditions similar to the ones obtained without SLM, we followed the same procedure in identifying the writing parameters, both with SLM and with single focus. We explored the fabrication window by changing the average power identifying the highest writing speed at which the geometry of the produced structure did not show any deformation with respect to the designed one. At higher speed we could not observe a significant time saving, since the desired speed is never reached in the continuous back and forth movement over a rather short distance. We kept all the other parameters constant. In this way we identified the threshold powers for polymerization and for material damage. We then chose the writing power as the intermediate value between these thresholds. Being the material the same, we believe that this procedure allows us to achieve the same level of cross-linking and mechanical properties.

The Nichoid scaffold, shown in Fig. 6b, consists of three horizontal square grids of 90 µm side, vertically spaced by 15 µm. Each grid is composed by two perpendicular sets of six parallel lines. At each line crossing of the grids, a 30 µm tall column passes, to sustain them. Nichoids are then arranged in square blocks of 25 units, which means 5 Nichoids per side, with the side walls in common. The whole substrate is then covered by these blocks, separated by 30 µm corridors. We already demonstrated in our previous works how this geometry is particularly indicated not only to promote cell proliferation, like cellular scaffolds usually do, but also to maintain stem cells pluripotency, through the isotropic forces exerted by the scaffold on the cells, thanks to the accurately tailored pore size and geometry^13,33^. We tested two approaches to parallelize the fabrication with multiple foci: fabricating many Nichoids at the same time, one for each focus, and using all foci to produce one Nichoid at a time.

With the first approach, we could fit in the objective field of view 4 foci at the corners of a 90 µm square, to produce 4 Nichoids simultaneously, without any phase mask change on the SLM during fabrication. With the second approach, we could fit a higher number of foci, up to 6 in the objective field of view, in order to simultaneously draw many lines of the same Nichoid. To fabricate a complete structure, however, we have to change the phase masks during fabrication. Two masks were employed, producing respectively 6 and 2 foci, for drawing 6 internal grid lines and vertical columns, and 2 external wall lines. Both these masks were also rotated in the orthogonal direction to produce the crossing grid lines and the other 2 external walls. With this configuration, it was possible to irradiate all the 25 Nichoid blocks with continuous 450 µm long lines, changing the masks only 4 times for each block, instead of 100 times (4 times for each of the 25 Nichoids of the block). Mask change was automatically performed by the control software, synchronized to the translation stages. In order to adjust the power needed for each foci configuration, we generated the masks for the 2 foci creating some additional spots next to the zero order position, where they can be blocked by a spatial filter. With this technique, the foci focused inside the photoresist in all configurations had the same power. The scaffolds have been fabricated with the same translation velocity employed in our previous single focus fabrications, 3 mm/s, which we found to be the optimal velocity for this kind of structures with our setup. More data about fabrication parameters are given in Table 1. A depiction of the foci patterns used can be found in Fig. 1.

The fabricated structures were inspected with a tabletop scanning electron microscope (Phenom PRO, ThermoFisher Scientific), in order to control the fabrication quality and to measure line size.

To evaluate the scaffold autofluorescence, we measured the fluorescence intensity distribution of the sample in the blue (360–461 nm), green (470–590 nm) and red (570–670 nm) channels in the visible light spectrum, filtering out the background noise. We used Nichoid samples without cells and we performed a z-stack fluorescence acquisition with a confocal microscope (Olympus FV10i, Japan) under usual observation conditions for live biological samples (37 °C and water immersed scaffolds). All data were analysed by using Excel and images manipulated with Fiji-ImageJ.

To validate the hypothesis that Nichoids fabricated through multiple foci do not influence stem cells functionality and viability, we assessed a Live & Dead assay on mesenchymal stem cells. We used commercial Petri Dishes (Corning Cell Culture Dish 35 mm × 10 mm, treated polystyrene) by specially modifying the bottom with a Nichoid circular glass substrate of 8 mm diameter, sealed with biocompatible Loctite AA 3321 glue (Henkel, Germany). Rat bone marrow mesenchymal stem cells (RBMSCs, Mario Negri Institute for Pharmacological Research) were seeded directly in the Nichoid at a density of 20 000 cells/cm^2^ and incubated for the whole experiment with α-MEM culture medium (Pan-Biotech, Germany) (20% FBS, 1% PS, 1% L-glut, 1% SP). To perform the Live & Dead Cell Viability Assay (Invitrogen, Thermo Fisher Scientific, USA) we measured the fluorescence emission of vital cells (green) and the one of dead cells (red) by using confocal microscopy, repeating the acquisition every 24 hours (after seeding) in Z-Stack (ΔZ = 1 μm), both for Nichoids produced by SLM and for single focus ones. We performed a statistical analysis by selecting n = 25 areas of interest for each time point and measuring the fluorescence intensity signal in Z-stack from the two channels. Average intensity values and standard deviations were computed, and the two groups were compared by using Student T-Test (p < 5%).

## Data Availability

The datasets generated during and/or analysed during the current study are available from the corresponding author on reasonable request.
